# Molecular characterization of cross-kingdom RNA interference in *Botrytis cinerea* by tomato small RNAs

**DOI:** 10.3389/fpls.2023.1107888

**Published:** 2023-03-08

**Authors:** Si Qin, Javier Veloso, Guido Puccetti, Jan A. L. van Kan

**Affiliations:** ^1^Laboratory of Phytopathology, Wageningen University, Wageningen, Netherlands; ^2^Departamento de Biología Funcional, Escuela Politécnica Superior de Ingeniería, Universidad de Santiago de Compostela, Lugo, Spain

**Keywords:** *Botrytis cinerea*, high-throughput sequencing, host immunity, small RNA, tomato

## Abstract

Previous studies have suggested that plants can modulate gene expression in pathogenic fungi by producing small RNAs (sRNAs) that can be translocated into the fungus and mediate gene silencing, which may interfere with the infection mechanism of the intruder. We sequenced sRNAs and mRNAs in early phases of the *Solanum lycopersicum* (tomato)-*Botrytis cinerea* interaction and examined the potential of plant sRNAs to silence their predicted mRNA targets in the fungus. Almost a million unique plant sRNAs were identified that could potentially target 97% of all fungal genes. We selected three fungal genes for detailed RT-qPCR analysis of the correlation between the abundance of specific plant sRNAs and their target mRNAs in the fungus. The fungal *Bcspl1* gene, which had been reported to be important for the fungal virulence, showed transient down-regulation around 20 hours post inoculation and contained a unique target site for a single plant sRNA that was present at high levels. In order to study the functionality of this plant sRNA in reducing the *Bcspl1* transcript level, we generated a fungal mutant that contained a 5-nucleotide substitution that would abolish the interaction between the transcript and the sRNA without changing the encoded protein sequence. The level of the mutant *Bcspl1* transcript showed a transient decrease similar to wild type transcript, indicating that the tomato sRNA was not responsible for the downregulation of the *Bcspl1* transcript. The virulence of the *Bcspl1* target site mutant was identical to the wild type fungus.

## Introduction

Plants are continuously exposed to fungal pathogens but they are usually able to combat these pathogens and remain healthy. To achieve this, plants have evolved an array of defense mechanisms. One of the most extensively studied defense mechanisms is conferred by host programmed cell death (PCD) at the host-pathogen interaction site, which can subsequently restrict the invasion by biotrophic and necrotrophic pathogens. However, PCD does not necessarily stop the invasion of necrotrophic pathogens effectively. By contrast, necrotrophic fungal pathogens take advantage of the induction of host cell death for their infection as they feed on dead tissue. Plant PCD triggers a series of reactions in the host, such as fortification of the plant cell wall (by callose and lignin), local production of pathogenesis-related (PR) proteins, phytoalexins and production of reactive oxygen species (ROS) (Veloso and van Kan, 2018). Despite the induction of all these defense responses in the host plant, it cannot successfully halt the colonization by necrotrophs. It is thus relevant to study plant defense mechanism(s) which can successfully control plant diseases caused by necrotrophic fungi.

A novel insight in plant defense mechanisms has emerged in the past 15 years, demonstrating that plant transgene-derived double-stranded RNAs (dsRNAs) could induce gene silencing by RNA interference (RNAi) in invading pathogens and pests (Baum et al., 2007; Nowara et al., 2010; Iqbal et al., 2020), in a process referred to as host-induced gene silencing (HIGS). The proof of concept for exploiting HIGS to control a fungal pathogen was demonstrated by Nowara et al. (2010), who expressed a set of double-stranded RNAs in (stably or transiently) transformed plants that would target *Blumeria graminis* genes that encode either effector *Avra10* or 1,3-β-glucanosyltransferases involved in haustoria formation. The successful deployment of HIGS suggested the occurrence of trafficking of RNA molecules from a plant into a fungus that could induce RNAi in the fungus, and thus HIGS can be considered as an example of cross-kingdom RNAi (ckRNAi).

In later studies, it was reported that plant endogenous small RNAs (sRNAs) can be naturally translocated to parasitic organisms and trigger silencing of genes in the invaders through RNAi. This phenomenon provided an example of the natural occurrence of ckRNAi, and it was proposed to be an effective host defense strategy by several studies. The first case was in the cotton-*Verticillium dahliae* interaction, in which the production of two cotton microRNAs (miRNAs) increased upon infection by *V. dahliae* and the miRNAs were translocated to the fungal hyphae where they silenced specific *V. dahliae* genes (Zhang et al., 2016). Cai et al. (2018) demonstrated that Arabidopsis can secrete exosome-like vesicles containing sRNAs which can be taken up by *Botrytis cinerea* hyphae at the infection site. This study described two small interfering RNAs (siRNAs) that selectively accumulate in fungal cells and are predicted to target *B. cinerea* genes that are involved in vesicle-trafficking pathways. A study by Hou et al. (2019) described that Arabidopsis produces increased amounts of secondary siRNAs upon infection by *Phytophthora capsici*. They observed that Arabidopsis mutants that are impaired in secondary siRNA biogenesis exhibited hyper-susceptibility to *P. capsici*. In several pathosystems, the naturally delivered host sRNAs have been proposed to contribute to plant immunity (likely) *via* silencing genes in the invading pathogens (Cai et al., 2018; Hou et al., 2019; Zhang et al., 2016). This process could contribute to plant defense against pathogens when the target genes that are silenced indeed have an important role in virulence or development of the pathogens.

We aimed to further explore the role of natural ckRNAi in defense of a model crop against a fungal pathogen. *Solanum lycopersicum* (tomato) is an important crop species that can be parasitized by many pathogenic microbes, resulting in considerable economic losses. The necrotrophic pathogen *B. cinerea* can infect different organs of the above-ground part of tomato plants, causing devastating damage for the crop during both pre- and post-harvest stages. *B. cinerea* was shown to be able to take up both sRNA and dsRNA molecules from the environment (Wang et al., 2016). The ability to take up exogenous sRNAs is one of the preconditions for the full function of RNAi triggered by sRNAs or dsRNAs present in the fungal environment. This feature of *B. cinerea* has been exploited in plant protection with spray-induced gene silencing (SIGS) by the application on plant surfaces of synthetic RNA molecules targeting genes involved in growth or in signaling required for virulence (McLoughlin et al., 2018; Nerva et al., 2020; Spada et al., 2021). Furthermore, a classical HIGS approach was also shown to enhance crop resistance against *B. cinerea* using transgenic potato lines expressing dsRNA that can target a *B. cinerea* gene which regulates cell growth and proliferation (Xiong et al., 2019). However, the efficacy of naturally occurring ckRNAi in the *B. cinerea*-crop interaction mediated by host-derived sRNAs is not fully conclusive.

In this study, we aimed to evaluate the role of endogenous sRNAs of the host plant in the early phases of the tomato - *B. cinerea* interaction. A sRNA and messenger RNA (mRNA) - sequence dataset were generated within the first 24 hours post inoculation (hpi). This dataset was described in a previous study (Qin et al., 2022), in which we could not validate the contribution of *B. cinerea* sRNAs to fungal virulence. We turned our focus to the inverse side of the tomato – *B. cinerea* interaction, namely tomato sRNAs and their function in plant defense against *B. cinerea via* ckRNAi during a natural infection. Correlations were studied between the abundance of three selected tomato sRNAs and the transcript levels of their matching fungal genes. In an attempt to experimentally validate the causal relation between the level of one tomato sRNA and its predicted single target mRNA in *B. cinerea*, we mutated a *B. cinerea* gene that was reported to contribute to fungal virulence and was predicted to be silenced by a single tomato sRNA. Substitutions in the target site neither altered the expression profile of the mutant transcript nor enhanced the fungal virulence by escaping the silencing effect by the tomato sRNA.

## Materials and methods

### Fungal strains, plant material and growth conditions

*B. cinerea* strains used in this study (**Table 1**) were grown and spores were collected as described in Zhang and van Kan (2013). Tomato (*S. lycopersicum* cv. Moneymaker) were grown in a greenhouse at 20°C for five to six weeks, and detached compound leaves were used for inoculation assays.

**Table 1:** *B. cinerea* strains used in this study.

*B. cinerea* strain	Description	Reference
B05.10	Wild type *B. cinerea*	van Kan et al. (2017)
*spl1-5mnt*	Strain containing synonymous substitutions of 5 nt in tomato sRNA target site in the *Bcspl1* coding sequence and a hygromycin-resistance cassette.	This study
*spl1-wt*	Strain containing wild type *Bcspl1* and a hygromycin-resistance cassette.	This study

### Tomato inoculations with *B. cinerea*

*B. cinerea* conidia were diluted in Potato Dextrose Broth (PDB, 12 g/l) medium to 1000 conidia/μl and 2 μl droplets containing the conidia suspension were inoculated on detached tomato leaves essentially as described by Zhang and van Kan (2013). The inoculated compound leaves were inserted in wet floral foam and incubated in closed containers at 20°C with relative humidity of ~100% under natural light, before being examined for the virulence assay or sampled for further purposes. For the virulence assay, each tomato leaflet was inoculated with three droplets on every leaf half for one *B. cinerea* strain. For the sampling of *B. cinerea* (or mock) - infected tomato leaves that was used for mRNA and sRNA sequencing, 10 droplets of 2 μl conidia suspension (or only PDB) were inoculated on both leaf halves at ~1 cm from the central vein. Four leaflets of one compound leaf were inoculated at the same time, and one leaflet was sampled by excising the central vein and collecting the remaining leaf tissue at each defined time point (t=0; 12; 16 and 24 hpi). For the sampling *B. cinerea* (or mock) - infected tomato leaves that was used for the quantitative reverse transcription PCR (RT-qPCR), the leaflets were inoculated in six to eight circular areas and area included five 2 μl droplets of conidia suspension (or only PDB). The inoculated areas were excised by a cork borer with a diameter of 15.6 mm at each time point (t=0; 12; 16, 24 and 36 hpi). More details on the inoculation and sampling design were described in Qin et al. (2022).

### RNA extraction

Fungal mycelium or tomato leaf samples were frozen in liquid nitrogen and used for extraction of small RNA using the mirPremier^®^ microRNA Isolation Kit (Sigma-Aldrich) while mRNA was isolated using the Maxwell^®^ 16 LEV Plant RNA Kit (Promega).

### Generation and bioinformatic analyses of the RNAseq dataset

Single-end Illumina sequencing was applied to all sRNA and mRNA samples by Vertis Biotechnologie AG (Martinsried, Germany) on a strand-specific library with read length of 75 nt. Sequence processing and bioinformatic analyses of data are described in Qin et al. (2022).

Differentially expressed genes (DEGs) in the fungus were identified by comparing fungal mRNA levels in the infected tomato leaf tissues with a *B. cinerea* liquid culture, as described in our recent study (Qin et al., 2022). Differentially produced sRNAs in tomato were analyzed by comparing tomato sRNA levels in the *B. cinerea-*infected tomato leaf tissues with the mock-inoculated leaf tissues at the same time point, as described in Qin et al. (2022). The thresholds for up- or down- regulation were calculated similarly for both mRNA and sRNA, using the DEseq2 algorithm through a negative binomial distribution to calculate the p-value. The significance was defined by thresholds that consisted of a p-value of lower than 0.05 and a log2 fold-change of higher than 1 or lower than -1 for up- or down-regulation, respectively. To determine the origin of the sRNA reads, the reads were mapped to both the *B. cinerea* and *S. lycopersicum* genomes. Bowtie was used as mapping tool with the constraint to only map reads without mismatches (-v 0). Reads that had a perfect match to the *B. cinerea* genome were also mapped to the *S. lycopersicum* genome and vice versa. Reads mapping perfectly on both genomes were labelled as ‘shared reads’ and were discarded, while reads that mapped to only one genome were labelled as ‘unique reads’. Target prediction of sRNAs was performed using sRNAs extracted from the ‘unique reads’. The tool psRNATarget was used to predict the plant sRNA targets on the fungal mRNA population (Dai et al., 2018), with settings adjusted to the default Schema V2 2017. The ‘expectation’ was set to match a free energy threshold of -20 kCal/mol (expectation 3). Both UTRs and CDS were tested for target sites. The sequences of the UTRs and CDS were obtained using the coordinates of the ASM83294v1 release-51 *B. cinerea* annotation. Predicted sRNA-target pairs were filtered by expression keeping only the sRNA-target pairs that showed sRNA up-regulation and target mRNA down-regulation.

### RT-qPCR quantification of mRNA and sRNA levels

Synthesis of cDNA from mRNA was performed using M-MLV reverse transcriptase (Promega). For reverse transcription of sRNA, the qScript microRNA cDNA Synthesis kit (Quanta Bioscience) was used. RT-qPCR was performed using SensiMix SYBR Hi-ROX Kit (Bioline). Primer combinations described in **Supplementary Table 1** were used in RT-qPCR to quantify levels of mRNAs and sRNAs. The transcript level of a ribosomal protein encoding gene *Bcrpl5* from *B. cinerea* was used to normalize fungal mRNA levels. A sRNA of the tomato U6 spliceosomal RNA component was used to normalize plant sRNAs. The threshold cycle (CT) values were determined by Bio-Rad CFX Manager 3.1 and fold-changes calculated using the 2^−ΔΔCt^ method (Pfaffl, 2006).

### *B. cinerea* transformation

*B. cinerea* mutant strains used in this study were generated by PEG-mediated protoplast transformation as described by (Kars et al., 2005) with minor modifications. The constructs of donor templates were made by the yeast recombination method as described by Schumacher (2012). For the construction of plasmids as well as the amplification of donor templates, PCR was performed with the primer sets shown in **Supplementary Table 1** using the Expand™ High Fidelity PCR System (Sigma). After obtaining transformed colonies on hygromycin-selective plates, the screening of transformants was performed by PCR with primer sets indicated in **Supplementary Table 1** using the GoTaq^®^ G2 DNA Polymerase (Promega). The coding region of the *Bcspl1* gene from the mutants used in this study was sequenced to verify whether or not the sRNA target site contained the 5-nucleotide substitution.

## Results

### *B. cinerea* genes down-regulated during infection and potentially targeted by sRNAs from tomato

Differential expression analyses of fungal genes were performed by comparing gene expression during infection of tomato leaves with a *B. cinerea* liquid culture, as described in Qin et al. (2022). More than 3000 *B. cinerea* genes (one quarter of the annotated genes) were differentially expressed at 24 hpi, as compared to growth in liquid culture, of which 1724 were up-regulated and 1282 were down-regulated (**Supplementary Data 1**). Among the up-regulated genes of *B. cinerea* at this time point of infection were genes encoding five polygalacturonases and 103 other Carbohydrate-Active enZYmes (CAZymes), 51 proteases, 63 membrane transporters, 5 proteins involved in signaling and 12 putative transcription factors. Moreover, the cluster of *Bcboa* genes encoding biosynthetic enzymes for the production of the polyketide phytotoxin botcinic acid were upregulated at 24 hpi, as compared to the *in vitro* liquid culture. Meanwhile, the *in planta* down-regulated genes (at 24 hpi) included genes encoding 36 CAZymes, 27 proteases, 39 membrane transporters, 33 proteins involved in signaling and 93 putative transcription factors. Moreover, there was significant down-regulation *in planta* at 24 hpi of melanin biosynthetic genes (Schumacher, 2016), of the gene cluster encoding biosynthetic enzymes for production of the sesquiterpene phytotoxin botrydial (Porquier et al., 2016), as well as the non-ribosomal peptide synthase gene *Bcnrps8* and six polyketide synthase genes along with their flanking genes involved in synthesis of (yet unknown) secondary metabolites (**Supplementary Data 1**).

Within the entire dataset, approx. 15% of the *B. cinerea* genes (1713 genes) that were predicted targets of the tomato sRNAs indeed displayed significant down-regulation in at least one of the time points (12, 16 and 24 hpi) as compared to the liquid culture (Qin et al., 2022). These fungal transcripts were, on average, predicted to each be targeted by 19.3 unique tomato sRNAs (**Supplementary Data 2**). The list of *in planta* downregulated genes that are predicted to be silenced by tomato sRNAs includes genes encoding 63 CAZymes, 43 proteases, 46 membrane transporters, 46 proteins involved in signaling and 101 putative transcription factors (**Supplementary Data 2**). Only 30 fungal genes were predicted to be targeted by a single tomato sRNA, including transcripts encoding fumarase BcFUM1, glutathione S-reductase BcGST8, catalase BcCAT7, phosphatidylserine decarboxylase BcPSD, melanin biosynthetic enzyme BcBRN2 and the cell death-inducing effector BcSPL1.

### Correlation between mRNA down-regulation in *B. cinerea* and levels of tomato sRNAs that are predicted to target them

After examining the *in silico* prediction of *B. cinerea* genes targeted by tomato sRNAs based on the sequencing dataset, we aimed to validate and establish in more detail the host sRNA – fungal mRNA profiles during the early infection. We performed new experiments to inoculate tomato leaves with *B. cinerea* and sampled at seven time points within the first 24 hpi. sRNA and mRNA were extracted from these samples, followed by quantification of the expression levels of selected tomato sRNAs (Sl-sRNAs) and their matching target mRNAs in *B. cinerea* (Bc-mRNAs) by reverse transcription-quantitative PCR (RT-qPCR). We selected three Sl-sRNAs and their predicted target Bc-mRNAs (**Table 2**) for molecular validation. The selection of sRNA-mRNA pairs was based on the following criteria: i) the predicted target mRNA showed sufficient reads in the sequencing dataset at all infection time points; ii) the predicted target mRNA was significantly down-regulated at one or more time point(s) as compared to the liquid culture; iii) the target gene might contribute to fungal infection; iv) the tomato sRNA was up-regulated in the early stages of infection and should be derived from a transposon locus. The following three *B. cinerea* genes were chosen for further analysis: the 5-oxoprolinase gene *Bcoxp1 is* a homolog of the *Fusarium graminearum oxp1* gene which was reported to be involved in development and virulence (Yang et al., 2018); the gene *Bccnd1*, encoding a secreted effector protein that is expressed in a calcineurin-dependent manner (Viaud et al., 2003) and is homologous to *GAS1* and *GAS2* effectors of *Magnaporthe grisea*, expressed in appressoria and required for full virulence (Xue et al., 2002); and the cerato-platanin gene *Bcspl1*, encoding an effector that induces plant cell death and is important for virulence (Frías et al., 2011).

**Table 2:** Selected predicted *B. cinerea* target genes and their corresponding Sl-sRNA sequences.

Annotation of target *B. cinerea* gene	Gene name^a^	ID^b^	Sl-sRNA – Bc-mRNA alignment	Free energy (kCal/Mol)
5-oxoprolinase	*Bcoxp1*	Bcin04g01040	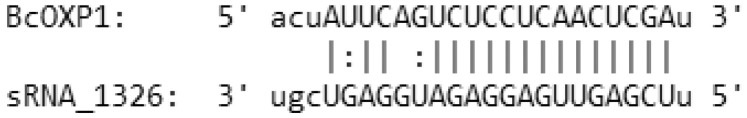	-34.51
Calcineurin-dependent (CND) gene	*Bccnd1*	Bcin08g05540	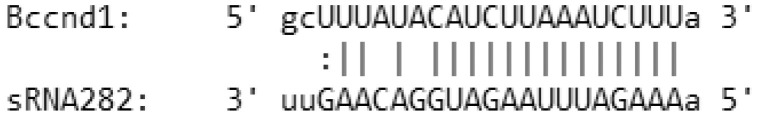	-20.19
Cerato-platanin	*Bcspl1*	Bcin03g00500	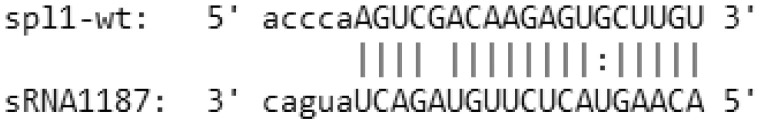	-28.41

**Figure 1:**
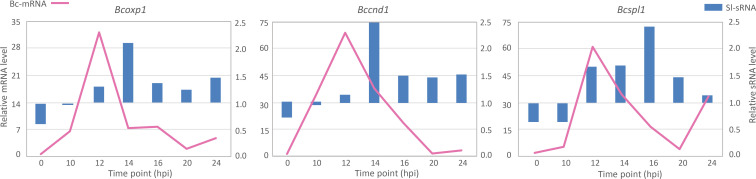
Quantification of production levels of three Sl-sRNAs (blue bars) and mRNA levels of their predicted target genes in *B*. *cinerea* (pink lines). The expression data were collected from four experiments, and the predicted target *B*. *cinerea* gene is indicated above each chart.

Molecular quantification results from RT-qPCR indicated correlations between Sl-sRNAs and their matching Bc-mRNA targets (**Figure 1**). Expression levels of the three tested *B. cinerea* genes displayed upregulation at 12 hpi, and they reached their lowest level at 20 hpi but increased again at 24 hpi. The down-regulation of the three Bc-mRNAs coincided with, or followed shortly after, an increase of the levels of their corresponding Sl-sRNAs. Specifically, the lowest expression of the three Bc-mRNAs occurred at 20 hpi, while Sl-sRNAs levels were high at 14 hpi or 16 hpi (**Figure 1**). Interestingly, levels of these three selected Sl-sRNAs all displayed an approximate doubling in the early stage of fungal infection between 12 and 14 hpi, or between 14 and 16 hpi.

### Generation of *B. cinerea* mutant with synonymous substitutions at the target site in *Bcspl1*

**Figure 2:**
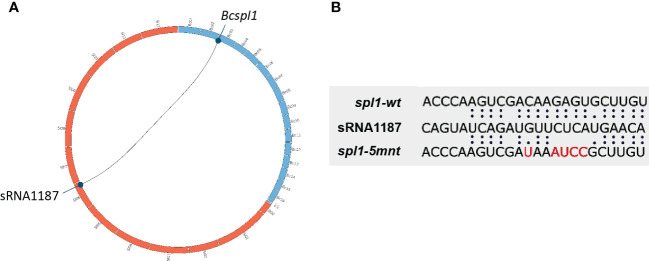
**(A)** Representation of the tomato genomic locus producing the sRNA1187 (tomato chromosomes presented as orange boxes) and the *B*. *cinerea Bcspl1* locus (*B. cinerea* chromosomes are presented as blue boxes). **(B)** Alignment of the sequences between tomato sRNA1187 and the wild type *Bcspl1* transcript or the mutated version designed to abolish interaction with the sRNA.

From *B. cinerea* genes that were predicted to be targeted for silencing by tomato sRNAs, we selected one gene for experimental validation of a causal relation between the presence of the tomato sRNA and the down-regulation of its target *B. cinerea* mRNA. Selection of the gene was based on three criteria: its transcript should decrease at some time during infection, as compared to previous time point(s) in the dataset; the transcript should be (predicted to be) targeted by a single tomato sRNA, in order to minimize the impact of multiple sRNA-mRNA interactions; the gene should participate in virulence of *B. cinerea.* The *Bcspl1* gene was selected as it is the predicted target of a single tomato sRNA (sRNA1187) and encodes a cell-death inducing effector protein with a role in virulence (Frías et al., 2011). Only 30 genes were predicted to be targeted by a single tomato sRNA (**Supplementary Data 2**) and *Bcspl1* was the only gene in this list that had been reported to participate in virulence. During *B. cinerea* infection on tomato leaves, *Bcspl1* displayed a peak in transcript level at 12 hpi and was ~10-fold down-regulated at 20 hpi (**Figure 1**). The lower transcript level of *Bcspl1* coincided with a transient peak in the level at 16 hpi of the tomato sRNA1187 (**Figure 1**), which is produced from a transposable element on tomato chromosome 6 (**Figure 2A**).

**Figure 3:**
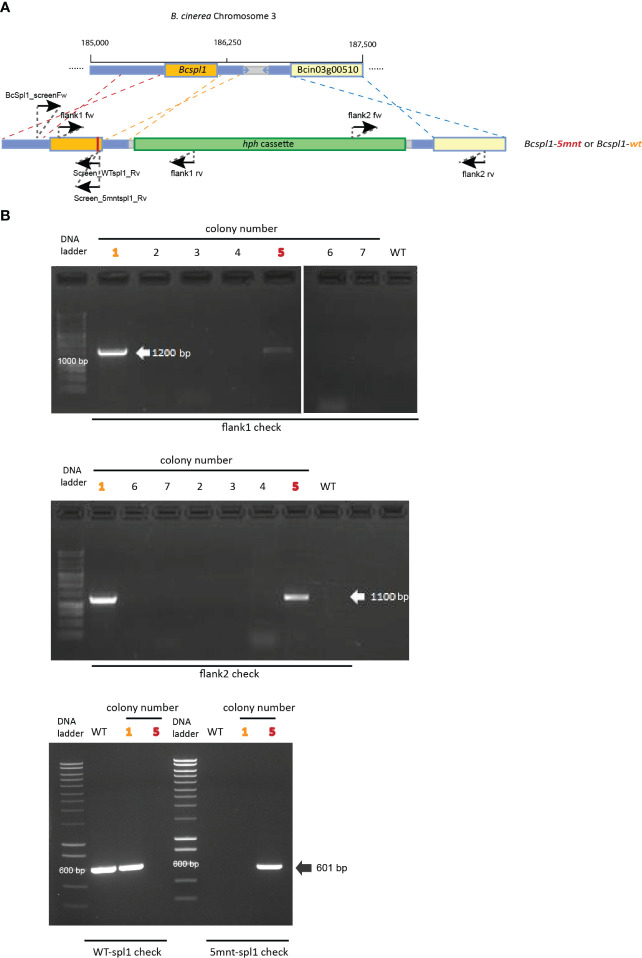
**(A)** Schematic representation of recombination events resulting in the generation of the transformants *spl1-5mnt* (red dashed lines) or *spl1-wt* (orange dashed lines). The blue dashed lines indicate the 3’-recombination event. The positions of primers used in genotyping the transformants are indicated. **(B)** Genotyping of transformants by PCR. Colony 1 (marked in orange) was the *spl1-wt* transformant while colony 5 (marked in red) was the *spl1-5mnt* mutant. Colonies 2-4 and 6-7 were ectopic transformants.

In order to disrupt the ability of *Bcspl1* mRNA to interact with sRNA1187, we aimed to generate a *B. cinerea* mutant carrying a *Bcspl1* allele with mutations in the sRNA target site. A substitution of five nucleotides would result in a change of free energy of the hybrid between sRNA1187 and the *Bcspl1* mRNA from -28 kCal/Mol (wild type) to -11 kCal/Mol (mutant), without changing the encoded protein (**Figure 2B**). The cut-off for free energy of RNA hybrids is -20 kCal/Mol (Marín and Vaníč, 2011), implying that this substitution would fully abolish sRNA-mRNA hybrid formation. We checked that the synonymous substitutions in the *Bcspl1* target site would not result in a sequence that could inadvertently be targeted by different tomato sRNAs in the dataset. A gene replacement construct was generated that encompassed the entire *Bcspl1* gene (containing the desired five-nucleotide substitution) and a part of the downstream gene Bcin03g00510 with a hygromycin-resistance cassette (*hph*) inserted in the intergenic region (**Figure 3A****)**. Transformation of this construct to wild type *B. cinerea* strain B05.10 yielded seven transformants of which two contained the *hph* cassette in the target locus and five were ectopic transformants (**Figure 3B**). The *Bcspl1* gene from both transformants was amplified and sequenced. Transformant #5 contained the desired substitution and was named *spl1-5mnt.* Transformant #1 contained a wild type *Bcspl1* sequence, presumably as a result of recombination with the target locus downstream of the sRNA target site (orange dashed lines in **Figure 3A**). This transformant was designated *spl1-wt* and served as a control transformant to exclude an impact on transcript levels caused by introducing a *hph* cassette close to the *Bcspl1* locus.

### Effect of substitutions at the sRNA target site on *Bcspl*1 expression profile

We hypothesized that if the tomato sRNA would indeed participate in silencing of *Bcspl1* in *B. cinerea* during infection, the synonymous substitutions in the sRNA target site would result in a distinct *Bcspl1* transcript profile during infection, i.e., the downregulation between 16 and 24 hpi (as observed in **Figure 1**) would be abolished. This might result in a higher virulence of the fungal mutant if the increased production of BcSPL1 protein would enable the fungus to trigger host cell death more effectively.

**Figure 4:**
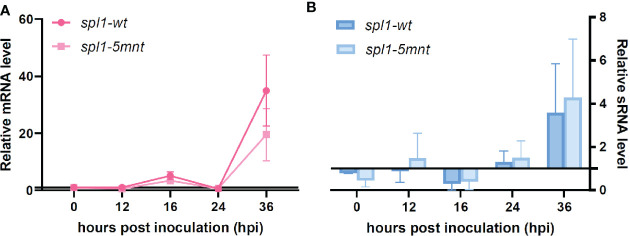
Expression levels of *Bcspl1* mRNA **(A)** and sRNA1187 **(B)** in *spl1-wt* and *spl1-5mnt* inoculated tomato leaves during the first 36 hpi. The expression data are shown by mean relative expression levels with standard error, either in a line chart **(A)** or a bar chart **(B)**, resulting from four independent inoculation assays.

**Figure 5:**
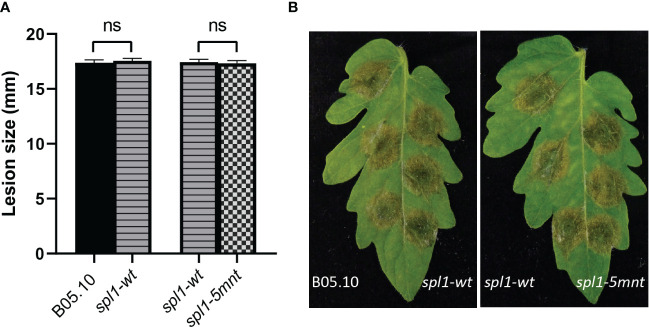
Virulence of *B*. *cinerea* WT B05.10*, spl1-wt* and *spl1-5mnt* transformants on tomato leaf, represented by a bar chart **(A)**; and disease symptoms photographed at 3 dpi **(B)**. The average lesion sizes and standard errors in the bar chart **(A)** resulted from 92 inoculations in three independent experiments, and statistical analysis was performed using t-test (ns indicates non-significance).

We inoculated tomato leaves with both the *spl1-5mnt B. cinerea* mutant and the control *spl1-wt* transformant and sampled the tomato leaf samples at four timepoints between 12 and 36 hpi. RT-qPCR was performed to quantify the *Bcspl1* expression in *spl1-5mnt* and *spl1-wt* mutants. Contrary to the hypothesis, the *Bcspl1* transcript profile in *spl1-5mnt* was similar to that in *spl1-wt* (**Figure 4A**), indicating that the downregulation of *Bcspl1* at 24 hpi was not abolished despite the substitution in the predicted target site for sRNA1187. Additionally, the level of tomato sRNA1187 was not significantly different between the leaf tissues infected by *spl1-wt* or *spl1-5mnt* isolates (**Figure 4B**). Infection assays were performed to compare the virulence of both transformants to each other and to wild type strain B05.10. As shown in **Figure 5**, there was neither a difference in virulence between *spl1-5mnt* and *spl1-wt*, nor between *spl1-wt* and wild type B05.10. These experiments did not provide any evidence for participation of tomato sRNA1187 in the downregulation of *Bcspl1* mRNA during infection.

## Discussion

In an earlier study (Qin et al., 2022) we could not identify any evidence for the contribution of *B. cinerea* small RNAs to virulence through the induction of ckRNAi during infection. There are several examples that individual small RNAs, as those produced by *B. cinerea*, can indeed silence plant genes when expressed in stable or transiently transformed plants at high levels, as reported by Weiberg et al. (2013) and Wang et al. (2017). In a natural infection of *B. cinerea* on a host plant, however, the amount of fungal biomass is very low as compared to plant biomass at early time points of the interaction. As argued by Veloso and van Kan (2018), the decisive processes that determine success or failure in the *Botrytis-*host interaction occur around 16 hpi. In this phase of the interaction, our RNA samples typically contained ~1% fungal RNAs (both for the sRNA and mRNA pools) in the entire dataset. Fungal hyphae that penetrate the plant surface and enter the interior plant tissues are vastly outnumbered by plant cells. Furthermore, the fungal sRNA population consists of thousands of distinct unique sequences and their potency in silencing target mRNAs in a host plant is essentially defined by random chance (Qin et al., 2022). If a specific fungal sRNA (at such low abundance) is able to target a plant mRNA and induce its silencing, it is unlikely that this will result in effective interference with crucial plant functions in the short time span of just a few hours. Indeed, our earlier study (Qin et al., 2022) showed that *B. cinerea* mutants in which both Dicer-like genes were deleted did not produce any detectable transposon-derived sRNAs, and could infect four distinct host plant species as effectively as the wild type of fungus.

One can envisage that the inverse situation (plant sRNAs targeting fungal mRNAs) is distinct, as the abundance of plant RNAs largely exceeds that of fungal RNAs. In an early phase of the plant-fungus interaction, fungal hyphae that penetrate the host surface and enter the plant tissue are surrounded by a large number of plant cells, each of which may produce and release small RNAs. *B. cinerea* can take up sRNAs present in exocytotic vesicles that accumulate at the extracellular interface between the plant and fungal cells (Cai et al., 2018). Fungal appressoria and invasive hyphae could have a high propensity to take up host plant exocytotic vesicles containing sRNAs.

During the *B. cinerea-*tomato interaction, numerous fungal genes were downregulated over the course of the infection process. Based on observations of Weiberg et al. (2013) and Wang et al. (2016), and the identification of 88,196 potential, predicted Sl-sRNA – Bc-mRNA interactions in our dataset (Qin et al., 2022), it was tempting to consider that many changes in fungal transcript levels were indeed caused by cross-kingdom RNAi. However, other explanations for down-regulation of fungal transcripts should also be taken into consideration. *B. cinerea* undergoes infection-related developmental transitions during penetration of the host tissue, by forming either appressoria or infection cushions, each with their specific developmental and transcriptional program (Leroch et al., 2013; Choquer et al., 2021). Once these infection structures have completed host surface penetration (10-14 hpi), they become redundant and the fungus switches to an intercellular hyphal growth while suppressing host cell death (Veloso and van Kan, 2018). From ~16 hpi, host cells are triggered to undergo programmed cell death and the fungus is exposed to oxidative stress in dying host tissue (Torres et al., 2006; Choquer et al., 2007). These developmental transitions and changes in chemical environment are likely to have a much greater impact on fungal gene expression than the presence of any plant sRNAs.

We identified three *B. cinerea* genes related to fungal infection that were predicted to be targeted by tomato sRNAs and did indeed display a transient reduction of mRNA level during infection, as compared to liquid culture. RT-qPCR analysis was performed with sampling at seven time points between 0 and 24 hpi, in order to establish an association between the level of the sRNA and its target mRNA with a high resolution of the dynamics. We observed an inverse correlation between the level of each selected Sl-sRNA and its predicted target in *B. cinerea*, which suggested that ckRNAi possibly could have contributed to achieving this downregulation. However, whether the production of unique plant sRNA molecules able to target a fungal mRNA could actually cause the downregulation of its target was difficult to establish merely from the expression profiles.

We therefore introduced mutations in the target site of a *B. cinerea* gene, *Bcspl1*, aiming to establish a causal relation between the production of the unique tomato sRNA molecule and the downregulation of *Bcspl1* mRNA in *B. cinerea*. The *B. cinerea* transformant with the allelic variant of *Bcspl1* displayed a similar transcript profile and the same virulence as the control transformant with an unaltered target site. This result indicated that the transient downregulation of the *Bcspl1* transcript at 24 hpi was not mediated by the tomato sRNA, but possibly controlled by an intrinsic regulatory mechanism within the fungus, instead of resulting from cross-kingdom RNAi. Only a single pair of Sl-sRNA – Bc-mRNA was examined in this study due to the restriction by the criteria that we set. In order to study in more detail whether tomato sRNAs indeed play a role in plant defense against *B. cinerea*, two aspects should be taken into account. Firstly, many sRNAs are derived from transposons, some with multiple closely related, but non-identical copies, and it is genetically impossible to dissect the function of individual naturally-produced sRNAs from the host. In order to functionally eliminate the vast majority of tomato sRNAs, one should use a tomato mutant in which multiple Dicer-like (*DCL*) genes are knocked out. There are seven *DCL* genes in tomato (*SlDCL1*, *SlDCL2a-d*, *SlDCL3* and *SlDCL4*) (Bai et al., 2012), and *SlDCL1* or *SlDCL3*-silencing mutant as well as single loss-of-function mutant of *SlDCL2b* or *SLDCL4* are available (Yifhar et al., 2012; Kravchik et al., 2014a; Kravchik et al., 2014b; Wang et al., 2018). Phenotypic characterization of these individual mutants proved that different SlDCL proteins are responsible for the production of different types of sRNAs. In order to abolish the biosynthesis of all sRNAs that potentially participate in cross-kingdom RNAi, multiple *SlDCLs-*knock-out mutants should be used. Such plants are, however, not available and research in Arabidopsis has shown that the deletion of multiple Dicer-like genes can have serious impact on plant viability and morphology (Bouché et al., 2006). Besides, the host sRNAs which are naturally translocated into the fungus are only a small proportion of the total host sRNAs generated by DCL proteins. Many plants sRNAs have regulatory functions on endogenous genes by RNAi and influence developmental processes or resistance to stress. Therefore, an enhanced susceptibility of plant mutants lacking *DCL* genes would not directly prove the function of plant sRNAs in plant defense *via* ckRNAi, due to the numerous physiological roles of the *DCL* genes.

The natural occurrence and function of the ckRNAi from both sides of the plant-parasite interaction is a relatively young research topic. A number of studies concluded that ckRNAi is a naturally occurring phenomenon. There were also observations that specific plant sRNAs can play a role in plant defense against pathogens, however, mostly through indirect methods by either overexpressing these sRNAs in plants (Hou et al., 2019) or knocking out their predicted target genes in the pathogen (Zhang et al., 2016). The technical and biological challenges that we discussed above clearly limit the capacity to directly prove the occurrence and importance of natural ckRNAi. It thus remains debatable to what extent the natural occurrence of ckRNAi mediated by endogenous host sRNAs contributes to effective plant defense.

## Data availability statement

The datasets presented in this study have been deposited in the NCBI database under Bioproject at www.ncbi.nlm.nih.gov/bioproject/ with accession number PRJNA496584. Raw sequence reads are deposited in the Sequence Read Archive at www.ncbi.nlm.nih.gov/sra/ project SRP166089 under accession numbers SRX4902781-SRX4902782, SRX4902789, SRX4902791-SRX4902800, SRX15228005-SRX15228010, SRX15231189, SRX15231190, SRX19002251 (sRNAs) SRX4902771-SRX4902780, SRX4902783-SRX4902787, SRX4902790, SRX15231192, SRX15231193, SRX19002219 (mRNAs).

## Author contributions

SQ, JV, and JK designed the study. SQ, JV, and GP performed the experiments. JV performed the bio-informatic analyses. SQ, JV, and JK interpreted the data and wrote the manuscript. All authors contributed to the article and approved the submitted version.

## Supplementary material

Supplementary Table 1 The list of DNA primers used in this study.

Supplementary Data Sheet 1 Differentially expressed *B. cinerea* genes during the infection of tomato leaves (sampled at 12, 16 and 24 hpi), as compared with a *B. cinerea in vitro* culture. The data contains four Excel sheets, which respectively provide all DEGs at 12/16/24 hpi *in planta*, and a summary sheet presenting the numbers of DEGs that are described to encode transporters, CAZymes, proteases, proteins involved in SM biosynthesis, signaling components and transcription factors (TFs) at different timepoints.

Supplementary Data Sheet 2 The data includes three sheets, i) the list of *B. cinerea* genes which were predicted to be targeted by tomato sRNAs, and the number of unique tomato sRNA sequences and merged sRNA sequences are shown together with their predicted matching single fungal gene; ii) the details of log2 fold-change and the adjusted p-value for each predicted target fungal gene which were significantly down-regulated at no less than one time point *in planta*, and iii) a legend that explains the headings in the columns of the former two sheets.
